# Mapping evidence of young people’s experiences of sexual aggression in the United Kingdom: A systematic scoping review protocol

**DOI:** 10.1186/s13643-020-01362-1

**Published:** 2020-06-03

**Authors:** Delarise Maud Mulqueeny, Jennifer Roberts, Senzelokuhle Mpumelelo Nkabini

**Affiliations:** 1 Discipline of Public Health Medicine, School of Nursing and Public Health, University of KwaZulu-Natal, Durban, South Africa; 2 Department of Social Science, Gender and Education, School of Education, University of KwaZulu-Natal, Room 01-032, 121 Marianhill Rd, Pinetown, 3605 South Africa

**Keywords:** Young people, Sexual aggression, Experiences, United Kingdom

## Abstract

**Background:**

According to the UK’s Office for National Statistics, England and Wales reported a 2.9% increase in sexual aggression cases (3.4 million females and 631,000 males) between 2009 and 2019. In Scotland, sexual aggression cases increased by 66%, with 40% of these sexual violations being perpetrated on individuals under the age of 18 years, while incidents relating to sexual misconduct in Northern Ireland increased by 21.0%, with only 41.2% of those cases being prosecuted. Acts of sexual aggression can have physical, emotional and mental consequences which predispose young people to subsequent short- and long-term mental and social disorders and comorbidities. Such consequences include feelings of guilt, shame, anger, experiencing post-traumatic stress disorders, antisocial behaviour, alcohol and drug misuse and dependency, confusion surrounding sexuality and sexually transmitted illnesses including the human immuno-deficiency virus. However, despite the societal, health, economic and educational implications for young people in the UK and increasing statistics, few studies address this scourge. Hence, the objective is to systematically map evidence of young people’s experiences of sexual aggression in the UK and identify literature gaps that could inform future research.

**Methods:**

The included literature for this scoping review is published peer-reviewed articles of all research designs; grey literature including governmental reports, policy statements, conference and media reports; and unpublished theses. Electronic searches of databases and search engines such as Embase, Google, Google Scholar, EBSCOhost, CINAHL, PubMed, Education Resources Information Centre (ERIC), PsycInfo, World Health Organization (WHO), media organizations, governmental and education departments and higher learning websites for published literature. Additional searches will include screening citations in reference lists of articles and perusing “Cited by” logs. All retrieved literature will be exported to an Endnote X9.2 library. Duplicate documents will be deleted prior to title screening commencing. An adapted Mixed Method Appraisal Tool (MMAT) will be independently used by two reviewers to ensure a rigorous study and quality assessment of all included studies.

**Discussion:**

This scoping review employs a mixed-method approach to map and select relevant literature and summarize and report on young people’s experiences of sexual aggression in the UK. Once the data is summarized, it could inform planning and policy pertaining to a safe and effective sexual health curriculum for all young people, assist with the development of effective strategies to reduce sexual aggression and guide future research.

## Background

The United Kingdom (UK), like other countries, has seen a spike in sexual aggression with societal, health, financial and educational implications [1, 2]. Sexual aggression in the context of this review refers to “a continuum of manifestations of unwanted sexual behaviours that cover all acts of unwanted sexual contact from sexual harassment up to and including rape” [1]. It also includes unwelcome sexual activity including fondling, oral, anal or vaginal intercourse experienced by males, females and non-confirming gender identities and sexualities between the ages of 10 and 18 living in England, Wales or Northern Ireland and Scotland [3–5]. According to the UK’s Office for National Statistics, England and Wales reported a 2.9% increase in cases involving sexual aggression (3.4 million females and 631,000 males) between 2009 and 2019 [6–8], with only 85,000 (0.5%) females and 12,000 (0.1%) males reporting being a victim of sexual misconducts, and the majority (83%) not reporting such incidents to law enforcement officials [9]. Furthermore, cases relating to sexual aggression in Scotland had increased by 66% with 40% of the sexual violations perpetrated on victims under the age of 18 [10], while cases relating to sexual misconduct in Northern Ireland had increased by 21.0%, only 41.2% of those cases were being prosecuted with 55.9% of the remaining cases not recommended for prosecution [11]. Acts of sexual aggression can have physical, emotional and mental consequences which predisposes young people to subsequent short- and long-term mental and social disorders and comorbidities [12]. Such consequences include feelings of guilt, shame, anger, experiencing post-traumatic stress disorders, antisocial behaviour, alcohol and drug misuse and dependency, confusion surrounding sexuality and sexually transmitted illnesses including the human immuno-deficiency virus (HIV) [13–18]. A reduction or the elimination of these acts could ameliorate the well-being and quality of life of young people including school, college and university students.

Social and cultural understandings of young sexualities are paramount in ensuring that policies and effective pedagogic interventions assist in informing young people about healthy, consensual sexual practices [19]. Hence, various humanitarian organizations strive to eradicate sexual aggression and the resultant psychological, emotional and physical impact on victims and survivors [20, 21]. Furthermore, these organizations also promote the United Nations Sustainable Development Goals (SDG’s), especially SDG 3: *Good Health and Well-being*; SDG 16: *Peace, Justice and strong Institutions*; and SDG 5: *Gender Equality*. These SDGs are relevant to this review as they influence community outreach and engagement, education curriculums and policies and the elimination of the *HIV* epidemic and *AIDS* as a global health challenge by *2030* [21].

Due to the increasing statistic of sexual aggression in the UK, we conducted literature searches to find primary studies, systematic and scoping reviews that addressed the topic. However, our searches revealed that although sexual aggression has increased globally, most research has been conducted in the United States of America (USA) and other high-income countries. Hence, to the best of our knowledge, some empirical studies have been conducted in the UK but no current systematic nor scoping reviews have answered this review’s research question [22–25]. A scoping review was chosen to map evidence on sexual aggression in young people living in the UK as this type of information synthesis methodology would comprehensively examine the range, extent and nature of research on a complex topic requiring extensively reviewing [26]. It would be limited to UK literature to explore how sexual aggression is positioned within this national culture. Additionally, it could further establish conceptual boundaries of current British cultural norms that can be embedded and reproduced in social and pedagogic practices. Moreover, it could support research goals and develop more nuanced understandings of how sexual aggression is affected by or influences notions of sexuality, power and agency in young people. The review could also reveal if societal, educational and cultural norms play a role in sexual aggression and advance future research on how best sexual aggression in these spaces could be eradicated. This could contribute to short- and long-term disruption to young peoples’ physical and psychological well-being and the findings can be utilized to critique and update pedagogic and governmental policies to accurately reflect the contemporary context of young people’s experiences of sexual aggression and the impact and consequences thereof. Additionally, this review will identify research gaps, the potential of conducting systematic reviews and reveal challenges to theoretical frameworks, current legislation and policies in relations to sexual aggression in youth.

In conclusion, the findings of this systematic scoping review will contribute to existent literature to assist researchers, educators, and policy-makers to address, reduce and potentially eliminate sexual aggression among young people. Moreover, the relevance of reviewing literature that addresses this population, context and concept (Table 1) could unsilence this enervative, chronic scourge.

**Table 1:** PCC framework

**P**opulation	“Young people” refers to males, females and non-conforming gender identities and sexualities between the ages of 10 to 18 and includes individuals in and out of school [3].
**C**oncepts	“Sexual aggression” in the context of this review refers to “a continuum of manifestations of unwanted sexual behaviours that cover all acts of unwanted sexual contact from sexual harassment up to and including rape”. It includes unwelcomed sexual activity (fondling, oral sex, anal or vaginal intercourse) [1, 4].
**C**ontext	The United Kingdom (commonly known as the UK or United Kingdom of Great Britain and Northern Ireland) is a country comprising of England, Wales, Northern Ireland and Scotland [5]
**Publication year range**	None
**Language**	All

### Objective

To systematically map evidence of young people’s experiences of sexual aggression in the UK and identify literature gaps that could inform future research.

## Methods

To map the range, breadth and extent of literature relating to a broad spectrum of literature including various types of evidence on sexual aggression in young people living in the UK, we chose a scoping review over other information synthesis methodologies [27]. The rationale behind our choice was that the topic is broad, and a scoping review was considered the most appropriate information synthesis method over a systematic review. This methodology would chart relevant literature, identify research gaps that could guide future research and systematic reviews surrounding the topic. Under the guidance of Arksey and O’Malley’s framework, Levac et al.’s enhancements and the 2015 Joanna Briggs Institute’s guidelines [28–30], this review will pursue a five-stage framework: (i) identifying the research question, (ii) identifying relevant studies, (iii) selection of eligible studies, (iv) charting the data and (v) collating, summarizing and reporting the results. Although this review is not registered as a priori, the Preferred Reporting Items for Systematic Review and Meta-Analysis Extension for Scoping Reviews (PRISMA ScR) will inform this review [31]. Additionally, the Preferred Reporting Items for Systematic Reviews and Meta-Analysis: Extension for Scoping Review Guidelines (PRISMA-P) will ensure this protocol adheres to rigorous standards [32–34].

### Identifying the research question

The use of the Joanna Briggs Institutes PCC (Population, Concept, Context) framework will ensure that the study selection adjoins the research question and the eligibility criteria [30]. Hence, the primary research question guiding this review is:

What evidence exists regarding young people’s experiences of sexual aggression in the UK?

The review sub-questions are as follows:

- What evidence exists on the types of sexual aggression experienced by young people in the UK?
- What evidence exists on the factors that contribute to young people experiencing sexual aggression in the UK?

#### Eligibility criteria

The Population, Concept, Context (PCC) framework (Table 1) will determine the eligibility of studies that address the research question and guide the selection process [30].

### Identifying relevant studies

This scoping review will include all study designs including qualitative, quantitative and mixed methods; interrupted time-series; randomized and non-randomized controlled and uncontrolled trials; before and after, analytical cross-sectional and observational studies inclusive of case-control and prospective and retrospective cohort studies published in peer-reviewed journals. Additionally, grey literature including government reports, policy statements, abstracts and reports from conference proceedings, unpublished theses and dissertations and media reports will also be sourced. Literature evidence will be sourced from electronic databases and search engines, humanitarian websites and education departments and institutions of higher learning websites. These include Embase, Google, Google Scholar, EBSCOhost, CINAHL, PubMed, PsycInfo, Education Resources Information Centre (ERIC), the United Nations International Children’s Emergency Fund (UNICEF) and the World Health Organization (WHO). Additional literature will be sourced from hand searches of the reference lists of articles and by perusing “Cited by” logs that address the topic. A research assistant will contact the corresponding author/s directly when relevant articles are electronically unattainable, to attain the same. A librarian who has expertise in sourcing literature on sexual aggression will assist with the searches. Furthermore, to ensure the feasibility of conducting this scoping review, a pilot search was conducted on one database by two reviewers using the keywords: “Sexual aggression”, “young people”, “experiences” and “United Kingdom” and the results thereof are found in Table 2 below.

**Table 2:** Pilot database search results

Keywords	Date of search	Search engine	No. retrieved
“Young peoples” “experiences” of “sexual aggression” in the “United Kingdom”	13 August 2019	EBSCOhost	355

### Study selection

The study selection process will be guided by the research question and the eligibility criteria. All studies considered for this review will be uploaded to an Endnote X9.2 electronic library by the research assistant once all duplicate studies are deleted. Thereafter, the electronic library will be shared with two screeners (JR, DMM) who will independently screen titles and abstracts of articles for relevancy and inclusion. A third screener (SMN) will resolve any disagreements by discussion. The full article screening will also be independently conducted in parallel by two screeners (JR, SMN) with DMM resolving any discrepancies during this stage. Although sources of evidence which do not meet the eligibility criterion will be disregarded, a record of such sources and the reasons for them being disregarded will be kept in a separate file to allow this study to be reproduced.

#### Inclusion criteria

The following criteria will ensure the inclusion of various sources of evidence:

- Peer-reviewed studies relating to sexual aggression and young people in the UK
- Studies containing all study designs including qualitative, quantitative and mixed methods; interrupted time-series, randomized and non-randomized controlled and uncontrolled trials; before and after, analytical cross-sectional and observational studies inclusive of case-control and prospective and retrospective cohort studies published in peer-reviewed journals
- Grey literature (government reports, policy statements, conference abstracts and reports, media reports and theses and dissertations)
- Articles and studies irrespective of date and language

#### Exclusion criteria

This scoping review will exclude:

- Literature that includes females and males younger than 10 and older than 18
- Studies conducted on young people outside the UK

**Fig. 1:**
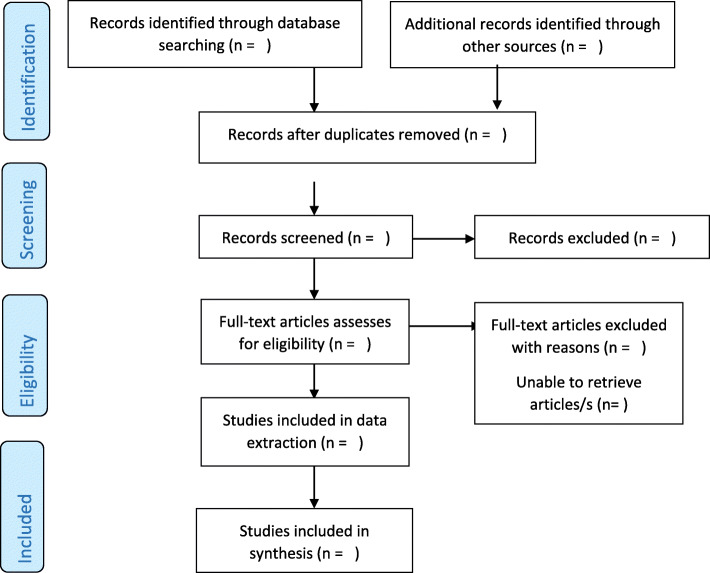
PRISMA ScR Flowchart demonstrates the literature searches and the selection of evidence process

The PRISMA ScR flow chart (Fig. 1) will capture and present a summary of the screening and inclusion and exclusion process [31].

### Charting of data

A data charting form was created using a Google form which will be populated with relevant information extracted from each included source of evidence. This process will be piloted by two assessors (DMM and SMN) to ensure that the data extracted is relevant and answers the research question and addresses the eligibility criteria. DMM and SMN will independently electronically populate the form with extracted data from each selected article. In the event of disagreements during this process, a third screener (JR) will intervene through discussion until consensus is reached. Due to the iterative nature of this process, the data extraction form (Table 3) will be continuously updated by the third screener to ensure it is current.

**Table 3:** Data charting table

Author and Year of publication	
Article Title	
Aim of the Study	
Study design	
Study setting	
Country	
Study population - Sexual Orientation - Gender - Age	
Characteristics of sexual aggression - Type of sexual aggression	
Key findings	
Theoretical Framework	
Data collection method	
Data Analysis Process	
Significant findings	
Conclusions	
Notes	

### Collating, summarizing, and reporting the results

The synthesis of the findings will be collectively described, coded, analysed and summarized by all the team members in relation to the study objective, the research question and eligibility criteria. Suggestions for future research based on the study findings will also be summarized and reported on. Discrepancies will be resolved by consensus between the primary author and reviewers throughout the process. Numerical counts and tables will summarize all quantitative data extracted, while thematic analysis will condense the qualitative data. These results will be presented in the PRISMA ScR chart. The results will identify literature gaps, implications for additional studies and systematic reviews and the implications for policy and practice.

#### Quality appraisal

To appraise the quality of each included source of evidence, avoid the risk of bias and ensure that the included sources of evidence meet the research objectives, the Mixed Method Appraisal Tool (MMAT) version 2018 will be utilized [35]. The determinants of the appropriateness of included sources of evidence are author and year of publication, appropriateness of the study aim, study design, study setting, country, study population, incidents of sexual aggression, data collection and analysis and the presentation of the study findings, discussion and conclusions. Additionally, the ratings of the MMAT tool (25% accounting for low-quality evidence, 50% accounting for average evidence, 75% being above average and 100% accounting for a high average score) will assist. Two screeners (JR, DMM) will appraise all the included sources of evidence.

## Discussion

Sexual aggression transgresses young people’s human rights with social, educational, physical, health and psychological short and long-term consequences [36]. However, despite this global human rights travesty and evidence indicating that sexual aggression among youth is a serious problem within the UK, to our knowledge, there is no overview of the evidence on this issue within the UK [37–39]. This study will map the breadth of available literature on sexual aggression among young people in the UK to inform future directions in research and policy on this issue within the social, cultural and political context of the UK. The review includes literature that addresses the population, context and concept (Table 1) and broadens the literature scope by not only including male-on-female sexual aggression but includes literature on sexual aggression in non-conforming genders and sexualities.

The proposed scoping review will map and summarize the extent, range and nature of research activities while noting various research approaches and methods to more accurately position a context to young people’s experiences of sexual aggression and their vulnerability in the UK.

Moreover, an exploration of sexual aggression can assist in identifying the types of sexual aggression, factors contributing to and the risk factors associated with these behaviours. The findings from this study will be presented at national and international conferences and could be used to inform pedagogic planning and policy, pertaining to a safe and effective sexual health curriculum for all young people in the UK. Additionally, it could assist with the development of effective strategies to reduce sexual aggression and HIV risk among young people and their partners and promote the United Nations SDGs 3, 5 and 16.

### Limitations

Conducting a scoping review with no time and language limitations could prove time-consuming and costly. Hence, strict timelines will be implemented to ensure the process is cost-effective and timeously completed.

## Data Availability

All data generated or analysed during this study will be included in the published scoping review article and will be available upon request.

## Abbreviations

HIV: Human immuno-deficiency Virus
UK: United Kingdom
USA: United States of America
WHO: World Health Organization
